# Investigating pre-professional dancer health status and preventative health knowledge

**DOI:** 10.3389/fnut.2023.1271362

**Published:** 2023-12-07

**Authors:** Joanna Nicholas, Sara Grafenauer

**Affiliations:** ^1^Western Australian Academy of Performing Arts, Edith Cowan University, Mount Lawley, WA, Australia; ^2^School of Health Sciences, Faculty of Medicine and Health, University of New South Wales, Randwick, NSW, Australia

**Keywords:** dance, low energy availability, REDs, nutrition, health, weight, menstrual disturbance

## Abstract

**Introduction:**

Dance is a highly demanding physical pursuit coupled with pressure to conform to aesthetic ideals. Assessment of health status and preventative health knowledge of pre-professional dancers may help inform educational strategies promoting dancers’ health and career longevity. The aim of this research was to establish a baseline understanding of dance students at a single pre-professional institution based on metrics focused on current health, nutrition, lifestyle, and wellbeing while also gauging knowledge of longer-term health implications.

**Methods:**

Adopting a cross-sectional study design, the Dance-Specific Energy Availability Questionnaire was tailored for Australian participants and administered online.

**Results:**

The response rate was 59.5% (69/116 eligible students) and the survey was completed in full by 63 students. Mean BMI was 20 kg/m^2^, although among females, 47% had a BMI < 20 (range 16 to 25 kg/m^2^), and at their lowest reported weight BMI was 14 to 25 kg/m^2^. Over a third had either experienced (31%) or were currently experiencing (3.4%) secondary amenorrhea (period absence ≥ 3 consecutive months). Most dancers did not exclude food groups, however, 24% had been advised to exclude particular foods in the past, mostly by dance teachers. A large percentage used nutritional supplements (68%) with 60% supplementing with iron and more than half (53%) taking two or more supplements. Only 25% had ever utilised a qualified dietitian, although 16% reported a history of eating disorders and 25% reported vegetarian or vegan eating patterns. REDs risk scores ranged from −16 to +16 points with negative scores indicating LEA and higher risk of REDs. The mean score for males was 5.2 (SD = 3.9) and 2.1 (SD = 5.9) for females, with 33.3% producing a negative score.

**Conclusion:**

Results provide insight to health knowledge and particular issues pertinent for dancers and highlights the need for specific education strategies to promote a preventative health focus for those entering a pre-professional programme. This study also highlights the need for improved awareness of LEA and REDs among all practitioners working with dancers along with cultural and structural changes within the broader dance community to help protect and promote the wellbeing of dancers.

## 1 Introduction

Dance training at a pre- and professional level is a highly demanding physical pursuit coupled with pressures for dancers to conform to aesthetic ideals. Rehearsal and performance volumes in professional ballet (mean 19.1 to 27.5 h per week and as high as 40 h per week) are much higher than training and competitions in elite sport and are likely to underpin end-of-season performance reductions and high rate of burnout among ballet dancers (1–3). While training demands are high in dance at a professional level (e.g., full-time role in a company), dancers training at a pre-professional level (such as at a full-time vocational or tertiary institution) are also faced with high volumes of physical training, along with juggling external stressors such as academic workload and living away from home for the first time (4).

Low energy availability (LEA) is defined as “any mismatch between dietary energy intake and energy expended in exercise that leaves the body’s total energy needs unmet, that is, there is inadequate energy to support the functions required by the body to maintain optimal health and performance” (5). High training and performance volumes in dance coupled with inadequate dietary intake can place a dancer at risk of low energy availability (6). Among dancers, there is reportedly a high prevalence of disordered eating (including excessive exercise in fear of gaining weight) and eating disorders (7, 8), restricting energy intake or engaging in excessive exercise can increase the risk of LEA. Recent studies have highlighted the importance of health and nutrition education among dancers to promote knowledge and awareness to potentially reduce LEA, disordered eating patterns, and injury risk while also assessing the supports needed for dancers entering into higher education (9, 10).

Despite LEA being first described in female dancers, the literature has been more broadly applied to other sports. Relative energy deficiency in sport (REDs) in particular, has gained the attention of the International Olympic Committee (IOC) and has recently been redefined as “impaired physiological and/or psychological functioning experienced by female and male athletes that is caused by exposure to problematic (prolonged and/or severe) low energy availability. The detrimental outcomes include, but are not limited to, decreases in energy metabolism, reproductive function, musculoskeletal health, immunity, glycogen synthesis and cardiovascular and haematological health, which can all individually and synergistically lead to impaired wellbeing, increased injury risk and decreased sports performance” (5). The key precipitator of this syndrome is LEA. The estimated prevalence of LEA/REDs in female athletes is 23 to 79.5% and in male athletes, 15 to 70% (5). These rates are across a wide range of sports with broad prevalence ranges due to inconsistent definitions and research methodologies (5). Previous studies in elite dance report 65% of female vocational (or pre-professional) ballet dancers (11), 40% of professional female dancers (12), and 57% female and 29% of male pre-professional, professional, and advanced amateur dancers (6) to be at risk of LEA or REDs. Among Norwegian jazz and contemporary university dance students, a reported 20 to 60% of dancers indicated symptoms of LEA, anxiety or depression, and/or symptoms of eating disorders and disordered eating behaviours (8).

Benchmarking health status and preventative health knowledge of those entering a pre-professional programme may help inform the required educational strategies to promote longevity in careers and optimal long-term health among dancers. Previous findings from a study among dancers suggest that the incidence of LEA and subsequent risk of REDs is higher (particularly among females) than awareness of these terms (6). Despite health and nutrition being highly rated by dancers in terms of interest, many pre-professional dance training institutions do not commonly include curriculum in these areas (10).

The aim of this research was to establish a baseline measure of students at a single Australian pre-professional institution based on a range of metrics focused on current health, nutrition, lifestyle, and wellbeing while also gauging knowledge of longer-term implications. The opportunity was to obtain insights for educational opportunities of dancers more generally, which could also be applied in younger age groups of vocational dancers where patterns of behaviour are established.

## 2 Materials and methods

### 2.1 Study design

A cross-sectional study design was adopted. Data collection took place in September 2022 via a self-administered anonymous online survey. The survey was based on the Dance-Specific Energy Availability Questionnaire (DEAQ) developed by Keay et al. (6). The DEAQ has been developed and administered by Keay et al. (6) specifically for dancers aimed at measuring and understanding the prevalence of LEA (6) and forms a useful screening tool for assessing risk of REDs. The DEAQ draws on three validated questionnaires, the Low Energy Availability in Females Questionnaire (LEAF-Q), a Sports Specific Energy Availability Questionnaire (SEAQ-I), and the IOC REDs clinical assessment tool (REDs CAT), and includes questions addressing the recognised physiological indicators of LEA, other potential correlates with LEA such as injury or illness and a basic nutrition assessment (6). Questions in the DEAQ relating to key food groups and serves of dairy were adapted in alignment with the Australian Guide to Healthy Eating (13). Vaping was added to the question related to smoking. An additional question was added to separate out the number of days of dancing missed due COVID-19 from other illness.

The anonymous online survey was developed in Qualtrics XM Platform™ (Provo, UT, USA) and pilot tested with five external dancers. The final 15-min survey consisted of 62 questions and used an open and closed questionnaire design with free text, multiple-choice responses and Likert scale questions. The survey was divided into six parts: (1) brief demographic information and dance background; (2) anthropometric data and dance training; (3) medical history, medications, and lifestyle; (4) smoking status; (5) injuries and sick days; (6) general health and nutrition knowledge. Scored questions (20/62) included weight/height (for calculation of body mass index; BMI), hormonal indicators specific to males and females, limitations on dietary patterns (including key food groups), smoking status, injuries, sleep patterns, and control tendencies including past medical history of eating disorders. Two questions that were scored within the REDs risk score related to how controlling weight and controlling eating affects dancers. These were assessed on a scale from 0 (“doesn’t affect me”) to 6 (“affects me greatly”). See Keay et al. (6) for specific DEAQ questions and REDs risk scoring tool. Ethical approval for this study was obtained through HREC at Edith Cowan University (REMS NO: 2022-03575-NICHOLAS) and HREC at the University of New South Wales.

### 2.2 Participants and procedure

Participation was open to all dance students at the Western Australian Academy of Performing Arts (WAAPA) regardless of age or year of study. Students were enrolled in either a ballet or contemporary-focused full-time dance programme, however, they are trained in both dance styles as well as other genres. Participation was optional and voluntary, and not linked with academic attainment. Written parental consent was sought for all participants < 18 years. Participant consent was obtained from all participants via the survey tool. An online survey link was distributed to the whole cohort via standard student communication channels including online announcements and weekly meetings. Participants were able to complete the survey in their own time using the link provided, a studio space was also provided to students following weekly meetings.

### 2.3 Data analysis

Participant response data was exported from Qualtrics (Provo, UT, United States) to a Microsoft Excel™ spreadsheet (Version 16.53, Washington, DC, USA) for calculation of LEA and REDs score. Descriptive statistics (means, standard deviation, ranges, and frequencies) for quantitative data were calculated using IBM SPSS (Version 29.0, Armonk, NY, USA). A summary report of free text response was also generated by Qualtrics (Provo, UT, United States) to assist with the qualitative data analysis. Free text responses were reviewed and collated and where necessary, content analysis was conducted and assigned to reoccurring themes by one researcher and independently checked by the other.

## 3 Results

### 3.1 Participant demographics and dance background

The survey was attempted by *N* = 69 of 116 enrolled dance students, providing a response rate of 59.5%. The survey was completed in full by *n* = 63, providing a completion rate of 91%. Due to the independent nature of each question, all survey responses, inclusive of those partially completed, were included in the final analysis with varied participation numbers reflected in the results. The majority of respondents were female (88.8%, *n* = 56) and aged between 16 and 24 years (mean 19.24 years) with most (51.6%, *n* = 33) from the first-year cohort. Table 1 provides detailed demographic characteristics and dance background of study participants. There was a large range in the age when participants first commenced dance training, from 1 to 17 years (mean age 5.2 years), with a mean transition to full time training at 16.5 years (10 to 21 years of age).

**TABLE 1:** Demographic characteristics including dance background of participants.

Demographic variable	Mean ± SD	Count (%)
**Gender (*n* = 63)**		
Female		56 (88.8)
Male		4 (6.3)
Non-binary		2 (3.2)
Not listed or other		0 (0)
Prefer not to say		1 (1.6)
**Biological sex (*n* = 63)**		
Female		58 (92.1)
Male		5 (7.9)
Age in years (*n* = 67)	19.2 ± 1.4	
16–17 years		4 (6.0)
18–20 years		50 (74.6)
21–24 years		13 (19.4)
Age started dancing (*n* = 67)	5.2 ± 3.1	
1–5 years		46 (68.7)
6–10 years		18 (26.9)
11–17 years		3 (10.4)
Age transitioned to full-time training (*n* = 67)	16.5 ± 2.3	
**Current year of study (*n* = 64)**		
First		33 (51.6)
Second		17 (26.6)
Third		13 (20.3)
Fourth		1 (1.6)
**Prior residential location (*n* = 68)**		
Australian Capital Territory		5 (7.4)
New South Wales		9 (13.2)
Northern Territory		2 (2.9)
Queensland		5 (7.4)
South Australia		3 (4.4)
Tasmania		0 (0.0)
Victoria		7 (10.3)
Western Australia		37 (54.4)
Other/overseas		0 (0.0)
**Current main dance genres (*n* = 68)**		
Classical ballet		53 (76.8)
Contemporary/modern		64 (92.8)
Jazz/musical theatre		5 (7.2)
Hip hop/urban/commercial		1 (1.4)
Other (text entry–tap)		1 (1.4)

### 3.2 Anthropometric measures and activity exposure

Anthropometric and activity exposure data is displayed in Table 2. Self-reported body weight for males ranged from 68 to 85 kg and for females ranged from 43 to 73 kg. Mean body mass index (BMI) for all participants was 20.5 kg/m^2^ (within the healthy weight range). Mean BMI for males was 23.3 kg/m^2^ (range 19.9 to 26.7 kg/m^2^) and for females was 20.2 kg/m^2^ (range 16.5 to 25.3 kg/m2), with 47% of female dancers reporting a BMI < 20. Participants were also asked to report their lowest body weight for current height. At their lightest weight, BMI for female dancers ranged from 14.6 to 25 kg/m^2^ with 61% reporting a BMI < 20.

**TABLE 2:** Anthropometric measures and activity exposure.

	Biological sex		Combined	
	Female (*n* = 58)	Male (*n* = 5)	Mean ± SD	Range (min–max)
**Anthropometrics**				
Height (m) (*n* = 63)	1.67 ± 0.68	1.78 ± 0.10	1.68 ± 0.1	1.45–1.9
Weight (kg) (*n* = 53)	56.3 ± 7.2	73.8 ± 6.7	58.0 ± 8.7	43.0–85.0
BMI (kg/m^2^) (*n* = 52)	20.2 ± 1.9	23.3 ± 2.8	20.5 ± 2.2	16.5–26.7
Weight variability (%) * (*n* = 51)	14.6 ± 6.8	10.1 ± 5.8	14.5 ± 7.0	4.0–38.0
**Activity exposure (hours) (*n* = 65)**				
Class	21.8 ± 6.1	18.0 ± 4.5	21.1 ± 6.5	0.0–40.0
Rehearsal	6.8 ± 3.3	6.0 ± 2.9	6.7 ± 3.3	0.0–15.0
Performance	1.5 ± 2.5	1.0 ± 2.2	1.4 ± 2.4	0.0–10.0
Supplemental training	2.9 ± 2.2	1.8 ± 2.7	2.8 ± 2.3	0.0–10.0
Total hours	33.0 ± 8.8	26.8 ± 2.0	31.9 ± 9.4	0.0–53.0

*Weight variability calculated for current height by dividing the difference between maximum and minimum weight by current weight, then multiplied by 100.

When asked about how often they weighed themselves, most dancers reported they did not weigh themselves (62.3%). Of those who did weigh, the mean frequency was 4.8 days/week and 17.4% reported they weighed themself less than weekly. Very few reported being weighed at a previous studio (*n* = 2/69), with weigh-in frequency being weekly. More than one-third of dancers (*n* = 24/69) had been told to lose weight at some point prior to commencing at WAAPA, mostly by past teachers.

When asked about what weight they dance best at, 34% either stated their current weight or provided the same numerical measure as their current weight, whereas 62% stated a lower weight from 0.5 to 7 kg lower than their current stated weight. Two dancers provided a higher weight in comparison to their current stated weight. Linked with this, 62.5% believe a lower weight was more likely to help them attain a leading role. Almost a third of dancers (28%) dancers reported not knowing or not weighing themselves and therefore did not provide a response to this question.

Participants reported dancing an average of 21.4 h per week (range 5 to 40 h), the large range in volume may have been impacted by dancers who were injured and not dancing at the time of data collection. Additionally, training and performance requirements differed greatly between cohorts at the time of data collection. Most dancers (81.5%) reported doing additional supplementary fitness or cross training for an average of 2.8 h per week (range 1 to 10 h).

### 3.3 Hormones and menstrual cycle

Male dancers made up a small portion of respondents (*n* = 5), all reported morning erections, mostly 3 to 5 days per week.

Among females (*n* = 58), menarche was reported by 67.2% between the ages of 12 and 14 years, with 24.1% delayed until after ≥ 15 years (primary amenorrhea). No dancers reported not having commenced menstruation. Over one-third of dancers (34.5%) had experienced oligomenorrhea (i.e., less than 9 periods per year), with two dancers (3.4%) currently experiencing secondary amenorrhea (i.e., periods stopped for three or more consecutive months, not due to pregnancy or contraceptive pill) and 31% experiencing this previously. Only 35% of dancers reported that healthcare practitioners had asked about periods, and this was only reportedly addressed in 42.1% (*n* = 8/19) cases where lack of periods were reported.

The oral contraceptive pill was taken by 27.6% of female dancers at the time of the survey, mostly for contraception (93.8%), however, reduction of menstrual pain, bleeding, and regulation of periods were also considerations. An equal number of dancers (46.6%, *n* = 27/58) knew that the hormones in the contraceptive pill were not equivalent to the hormones produced by the body, as those that did not know. A very small number (6.9%, *n* = 4/58) believed hormones produced by the body were equivalent to that provided in contraceptives.

When asked about dance health knowledge and beliefs (*n* = 63), 18.8% perceived it to be normal for female dancers to not have periods (i.e., menstruate) and 12.7% “did not know.” Furthermore, 11.1% were not aware of any negative effects of not menstruating and 7.9% did not know of any associated problems (*n* = 5).

### 3.4 Illness and injuries

Seventy percent (*n* = 44/63) reported sustaining a soft tissue injury in the past year (i.e., muscle, ligament, tendon, and joint injuries excluding fractures). There was an average of 1.6 injuries per dancer in the preceding year, 37% of dancers did not miss any days due to injury, and a further 35% missed ≤ 5 days. There were a small group of dancers (11.3%, *n* = 7/62) who had missed significantly more days of dance due to injury (28 to 210 days). Of the soft tissue injuries, 54% of participants reported that they were recurrent (i.e., in the same location, or same type of injury).

Over a third (34.9%, *n* = 22/63) had experienced a bone injury since starting full-time dance training. Where dancers had experienced a fracture, the most prevalent anatomical regions were the feet (28.6%, *n* = 18), followed by arm (17.5%, *n* = 11), leg (7.9%, *n* = 5), and spine (4.8%, *n* = 3).

General illness resulted in an average of 9.9 days absence affecting 85% of the cohort, and COVID-19 a further 12.2 days. Ten dancers had not experienced COVID-19 at the time of data collection.

When asked about missing classes or rehearsals, 65.2% of dancers felt “worried or anxious” to miss a class or rehearsal, 23.2% felt “relieved to have a day to rest,” 30.4% indicated they “understood these things happen.” A small number of dancers expressed feeling guilty, lazy, self-critical, judged, and expressed feelings of self-hatred.

### 3.5 Eating habits, supplements, and nutrition advice

Dancers were asked about particular dietary choices, with 21.9% (*n* = 14/63) reporting they chose a vegetarian dietary pattern, with just two (3.2%) selecting vegan. Almost half (43.7%) reported excluding food groups. Excluded food groups or categories included: carbohydrate foods (1.6%, *n* = 1/63), meat (20.6%, *n* = 13), fish (19.0%, *n* = 12), dairy (11.1%, *n* = 7), and gluten (9.5%, *n* = 6). Nearly one-quarter of dancers (23.8%, *n* = 15/63) had been told to exclude particular foods by teachers in the past, with a range of advice provided:

“*In the past it had been suggested for me to only eat light salads, and cruskits with cottage cheese to lose weight*.”

“*I’ve been told many things in the past, e.g., only eat raw foods as cooking reduces nutritional value, try an [Emma] Ware fast diet as it cleanses the gut*.”

“*Meat, dairy and bread. ‘To avoid bulking up’*.”

“*Sugary foods, carbs, fats, dairy, gluten*.”

“*Carbohydrates, fats, any processed foods*.”

With dancers noting that the advice was aimed at weight concerns:

“*For body image*”; “*To lose weight*”; “*Was told to cut carbs to reduce puffiness in body*”; “*Don’t eat/drink sugary things, you will be fat. Don’t eat lunch before class, the teacher says she can see our lunch*.”

Caffeine as caffeinated beverages were consumed by 58% (*n* = 39/67). The mean consumption was 1 cup per day (range 0–4 cups per day). Dairy consumption was varied with 42.2% consuming 2–3 serves/day, 12.6% (*n* = 8/63) consuming no dairy, and 51.6% consuming below the recommended serves/day (i.e., 0–1 serves/day). Supplements were used by 68% of participants, mostly iron supplements (60%) and magnesium (51%), with fewer (14%) taking calcium, zinc, Vitamin C, or a multivitamin. Vitamin B was taken by 19% and Vitamin D by 12%. More than half (53%) were taking more than two supplements and a list of 12 other supplements were provided.

The most common sources of nutrition advice in descending order were the internet (42.2%), the advice of friends (28.1%), and 25% sought advice from a qualified dietitian. Almost one-third (29%) had not sought any form of nutrition advice despite this same proportion had been told to lose weight at some point in their dance training. A follow-on question about the impact of social media was also asked, with 81.3% of dancers reporting that social media makes them feel like they should “sometimes” or “always” try and lose weight.

Five dancers reported smoking (7.9%, *n* = 5/63) for social reasons, stress management, or due to addiction. Three out of five reported trying to stop.

### 3.6 Wellbeing and psychological factors

A range of questions were asked to gauge wellbeing among the dancers. Dancers were asked to rate their freshness or energy level on a scale of 0 (“extremely tired all the time”) to 5 (“no fatigue at all”), with 84.4% providing a score ≥ 2 indicating higher levels of energy with two dancers reporting they were extremely fatigued. Similarly, when asked about sleep, most (64%) provided a score ≥ 3, with just three dancers reporting they hardly ever had a good night’s sleep in the past year. When prompted about sleeping difficulties, the key issues were difficulty falling asleep, disrupted sleep, and waking too early.

Digestive problems were also rated, with a small number of dancers (6.3%, *n* = 4/63) indicating continuous problems, usually linked with medical issues. Further prompt questions revealed bloating (66.6%, *n* = 42), constipation (33.3%, *n* = 21) and discomfort (50.8%, *n* = 32) were the top ranked issues among dancers with 11.1% (*n* = 7/63) reporting no such issues. Allergies and intolerances were reported by 40.6% of dancers, which may be linked with the issues regarding digestion, the core foods excluded, and supplement use described earlier. Medications were reported by 27%, with antidepressant used by 10.5% (*n* = 7) of the participants.

Ten dancers (15.6%) had previously been diagnosed with an eating disorder, including four with anorexia nervosa, two with bulimia, one reported a combination of anorexia nervosa and bulimia, another reported avoidant/restrictive food intake disorder, and another was “not specified.” We noted that one additional participant did not respond to the question regarding type of eating disorder.

### 3.7 Awareness and REDs risk score

REDs risk scores were calculated for 49 participants (45 females). REDs risk scores ranged from –16 to +16 points with negative scores indicating LEA and a higher risk of REDs. The average score for the cohort was 2.1 (SD = 5.9) for females and 5.25 (SD = 3.9) for males, indicating males in the study group were not at risk. Negative scores (< 0) were seen in female dancers with 33.3% scoring in the negative range.

Just over half of all participants were aware of REDs (53.1%) and female athlete triad (56.3%), and a large proportion acknowledged an awareness of LEA (73.4%). There was a high degree of awareness about disordered eating (92.2%) and just over a third were aware of orthorexia (35.9%).

The REDs risk score included questions about how controlling weight and controlling dietary intake affects dancers. These questions utilised a scale as described from 0 (“doesn’t affect me”) to 6 (“affects me greatly”), while 17.5% reported no issues, 60.3% of dancers rated controlling what they eat as 3–5/6, and no dancers gave a rating of 6/6. Controlling weight was more problematic, with 47.6% scoring 3–5/6 and an additional 20.6% scoring this question 6/6 indicating it “affects me greatly.” Control of body weight was of no concern for 24% of participants (*n* = 15/62).

## 4 Discussion

This study was based on the DEAQ by Keay et al. (6), a tool combining three validated questionnaires to better capture information relevant to dance, with a REDs scoring tool embedded. The current research was conducted with dance students at a single training institution to help understand current health status, nutrition habits, lifestyle, and wellbeing, while also gauging knowledge of LEA and REDs to inform specific curriculum related to dancer health and wellbeing. A third of female dancers in this study received a negative REDs risk score indicating low energy availability and risk of developing REDs. No males scored negatively, noting that males made up just 6.3% of the participant group. The risk of LEA and REDs among dancers in this study was less than reported in previous studies among elite dancers: 65% of female vocational ballet dancers (11), 40% of professional female dancers (12), and 57% female and 29% of male pre-professional, professional, and advanced amateur dancers (6).

Dance, particularly ballet, as a physical pursuit is unique. Exertion within a typical ballet class builds gradually over an hour to several hours from almost stationary movements at the barre, to grand allegro, with intermittent bursts of athletic jumps and sequences using the entire studio space or length of the room (14). A performance may last 3 to 7 min (14), and performers within a full ballet cycle on and off stage, or to the outer rim of the stage area, holding postures for extended periods of time. Performances involve high volumes of plies, lifts, and jumps eliciting near-maximal metabolic responses (3). With the metabolic demand of ballet classes being less than in performance, it is unlikely that ballet training alone provides metabolic and musculoskeletal adaptations (3). In contemporary dance, dance classes and rehearsals alone are reportedly inadequate in preparing dancers for the cardiorespiratory demands of performance (15). Although elite dance training involves high volumes of activity, professional ballet dancers spend half of recorded day-to-day activity at below 3 METs (metabolic equivalent of task) which is less than a moderate level of exercise intensity (3, 16). As dance classes alone are unlikely to induce adequate metabolic, musculoskeletal, and cardiorespiratory training effects, it is recommended dancers incorporate supplementary fitness training into their schedule (3). Supplementary fitness training can help promote strength, balance, proprioception, and cardiovascular endurance to promote fatigue-resistance and reduce injury risk (10, 17). Despite this suggestion, ballet training is intensive, beginning at a young age, most often as children begin school, as was typical within this study. Young vocational dancers may attend classes daily, with pre-professional dancers taking classes “full-time” 9 am to 5 pm on up to 5–6 days per week from about 16 years of age, with 20 h (on average) up to 40 h a week reported in this study, leaving very little time for the suggested supplementary training or food consumption between classes (18). Managing nutrient requirements within a tighter dietary energy intake when there is discomfort while dancing, or while being lifted, may further encourage dancers to limit food intake (food volume and/or kilojoules) to maintain a lower body mass and body fat percentage (14, 18). As a result, lower energy availability may become chronic, and malnutrition may also become an issue. In a recent study among Australian elite dancers (19), the reported average daily energy intake was lower than the typical intake reported in studies of elite dancers from the UK and Scotland (11, 18) and in particular, lower carbohydrate intakes. Furthermore, over an 8-week period a decline in iron (sFER) levels was observed in these dancers which was attributed to lower than adequate energy intake (19).

In pre-professional dance programmes, nutrition education may be absent or limited (20), unlike in elite sporting contexts other athletic sports programmes where nutrition education and qualified sports dietitians may be more accessible (21, 22). Only 25% of participants had sought individualised advice from a qualified dietitian, despite many following vegetarian (22%) and vegan (3.2%) eating patterns. This alteration in dietary pattern places an even greater focus on adequate energy intake, adequacy of iron, zinc, Vitamin B12, calcium, and omega-3 due to plant sources having reduced bioavailability (particularly iron) and insufficient plant-based B12 sources (18). It is therefore important for dancers to have the required knowledge, skills in planning, and perhaps professional guidance to ensure adequate intake enough of the nutrients that are more easily consumed through animal food sources. The number of dancers following non-meat diets also far exceeds that within the general Australian population with representative studies suggesting 4.3% of adults are vegetarian and 1.6% are vegan (23). As a consequence, meat and fish were the most frequently excluded food items. Additionally, more than half of the dancers in this study were not consuming adequate serves of dairy per day thereby leading to reduced calcium intake from food sources. Supplements were widely used, with more than half taking multiple nutrition supplements, mostly iron and magnesium. Fewer were taking Vitamin D supplements, yet this vitamin may be at low levels primarily due to lack of exposure to sunshine and the extended time spent exercising and training inside, rather than outside (14). There are recommendations for dancers in the northern hemisphere to monitor Vitamin D status and use supplementation (24), however, this would need further investigation among Australian dancers.

Nutrition education is included in the curriculum at WAAPA, focused on building awareness of the Government guidance regarding the Australian Dietary Guidelines, the Guide to Healthy Eating and serves of foods. Dance and sport-specific evidence-based resources such as publications developed by the International Association for Dance Medicine and Science and the Australian Institute of Sport are also emphasised. Several studies have evaluated the importance of incorporating health and nutrition education into elite dance programmes with improvements in health, nutrition, and food literacy reported (20, 25, 26). Despite nutrition education being delivered at WAAPA, many participants reported consulting the internet or friends for advice, and there was a high prevalence of dancers feeling like social media made them feel like they should lose weight. These are important considerations for future curriculum design. Due to the higher risk of LEA and REDs in dancers, Civil et al. (11) suggest that a qualified sports dietitian is involved with dance programmes to enhance knowledge, and personalise advice that is safe and sustainable. This may be even more useful if students are screened for LEA and REDs on admission and annually as a general health check, as there are known challenges transitioning to higher education (10). An understanding of those dancers most at risk at the pre-professional level could help prevent shorter term issues with performance, illness and injury, but also help protect longer term health issues particularly those of a reproductive nature, protection of mental health, and provide enhanced self-worth and assist with longevity of the dance career (27). Similar to the research by Civil et al. (11), further research related to the dietary patterns of this group would be valuable. Particularly in consideration of the vegan and vegetarian diet patterns where grain and legume foods are high in dietary fibre, but low biological value proteins, and also the tendency use supplementation which may be in place of nutrient-dense foods potentially further encouraging LEA. Dietitians were utilised by a quarter of the sample, however, their services could be beneficial to a larger portion of dancers by providing practical and individualised advice, assisting dancers make food choices that provide greater gastrointestinal comfort during dance training and performance (which was a common concern), maintain lean muscle mass, improve recovery, and help better match intake with rehearsal and performance schedules (11). Hypothetically, the provision of such services would normalise the acceptability of dietitians within dance, add significant promotional value for programmes offering this type of support, and form an ongoing important focus on health and safety. This is particularly important as poor knowledge and misinformation from dance teachers and internet sources could have a negative affect on dancers, including impacts on food selection and eating patterns.

Previous studies have found that dancer knowledge of LEA was understood, more so than REDs (6). Indeed, dancers in this study also indicated a greater awareness of LEA (73.4%) compared with REDs (56.3%). Perhaps the concept of having “low energy” available is more easily understood, as it can be related to day-to-day feelings of fatigue and tiredness, whereas REDs is the result of chronic and more complex interactions between dietary intake, hormonal, and bone health. Linked with an understanding of REDs, 19% of dancers in this sample perceived the absence of menstruation as normal for female dancers and were either unaware of negative consequences or any associated problems with a lack of menstruation. In dancer health training, it is worth educators or teachers explaining REDs to dance students, including REDs Health Conceptual Model and REDs Performance Conceptual Model proposed by Mountjoy et al. (5) along with raising awareness of symptoms such as amenorrhea, low libido, pain from bone stress injury, hunger, and training plateaus. Discussing the conceptual models and symptoms of REDs could provide an opportunity to discuss eating patterns, the known risks of amenorrhea or lack of morning erections for males, which can then be monitored by the dancer as a personal sign of health, along with longer-term problems associated with REDs such as osteoporosis. Importantly, under extreme circumstances, some of the negative consequences of REDs have proven difficult to reverse even with pharmacological means, placing even greater responsibility on the dance community to protect dancers of all ages. Of concern is the low level of awareness of menstrual disturbance and LEA among healthcare practitioners reported by dancers in this study. Despite a number of specific resources being developed for healthcare practitioners and the dance community (28) in recent years, there remains a lack of awareness among doctors (29).

In this study there was a reported 1.6 injuries per dancer in the past year, a similar finding to research among modern dancers [1.31 injuries per dancer (30)] yet less than in ballet [6.8 injuries per dancer (31)]. Soft tissue injuries were relatively common, with more than half being recurrent in nature. Results align with other research which reported soft tissue injuries [i.e., muscle and tendon inflammation (36%), and strains and sprains (27%)] as the most frequent injuries among a similar pre-professional group (*N* = 76) (8). Over one-third of dancers had sustained a bone injury since starting full-time dance, with the foot being the most common fracture site. These results align with previous research which indicates bone injuries in the feet are common in dancers (30, 32, 33). Previous research has identified that dancers with LEA are more likely to sustain a soft tissue injury and that training volume (exposure time) is associated with a higher risk of bone injuries (34). Similarly, in a study of Norwegian university dance students there was an increased odds of having an injury if symptoms of low LEA were prevalent (8). In another study, high training volume among professional ballet dancers was linked to insufficient bone remodelling, potentially leading to an increased risk of bone stress injury (35). It is well established that LEA contributes to impaired bone health (36), and previous research among dancers has highlighted the importance of adequate nutrition and controlled training volumes to optimise energy availability and reduce the prevalence of injuries (34).

In this study, 22% of dancers did not respond to the question about their body weight. This could be interpreted in a number of ways. Either they did not know or have not recently weighed, or they did not want to provide this information. Body weight was an important measure to estimate health status and was utilised within the REDs score to understand risk, but realising the issues among dancers and sensitivity to reporting weight, the data from this response may be highly unreliable. However, body weight data, together with the calculated BMI, was reflected in hormonal changes reported by this group with a third of dancers experiencing oligomenorrhea and 31% experiencing secondary amenorrhea previously. Dancers also reported their lowest body weight for current height with BMI calculations indicating as many as 61% of dancers having a BMI < 20 (below the healthy range) when they were at their lower weight. Sixteen percent of dancers had a previous history of eating disorders, which is within the prevalence range of 12 to 26.5% described in the literature across all dance genres (8). Future research should aim to understand the issues of weighing and weight, as there may be other factors involved. Rejection of body mass as a single measure is a point of discussion, and this is an important issue for the future of dance where rudimentary cut-offs have been applied for entry to competitions, programmes, and at the professional level in the past. The recently published 2023 IOC Consensus Statement on REDs (5) recommend avoiding body composition assessment and monitoring in athletes < 18 years, with the exception of medical purposes (37, 38). Further, the supporting Best Practice Guidelines highlight the importance of sport federations addressing competition requirements which perpetuate unhealthy practices around body composition and report multiple sports (including figure skating, gymnastics, and artistic swimming) modifying rules to reduce the risk of developing REDs associated with participation (38).

Other health concerns within this cohort can be drawn from information supplied relating to medication use, sleeping issues, digestive problems, and the way both eating and weight affect the dancer’s sense of self. There are known links between nutrition and mental health, depression and eating disorders, sleep disturbance, and physiological functions of the body such as digestive issues (39). Further, emerging adulthood has been highlighted as a key period of development in terms of both diet quality and mental health (39). Comparable studies of pre-professional dancers have reported 20 to 60% having anxiety or depression, symptoms of LEA and/or eating disorders (8). Importantly, one-third of dancers in this study ranked the questions relating to “controlling what they eat” and “controlling what they weigh” at the highest end of the scale (as 4 or 5 on a six-point scale), indicating that both factors affect the way they feel about themselves. Others have pointed to issues particularly related to dancers including “self-oriented perfectionism” whereby self-worth is highly linked with performance and feedback as well as the ability to push their body to artistic limits and constant improvement (8). Striving for perfectionism, however, can cause anxiety, stress, or compulsiveness which are reportedly potential comorbidities to eating disorders (40).

A large portion of dancers in this study reported the belief that a lighter body weight would increase chances of getting a lead role and many reported the belief that they dance best at a weight lower than their current weight. These findings align with previous studies reporting a desire for thinness and body dissatisfaction in dance (6), which are factors linked with an increased risk of disordered eating, eating disorders, and LEA (40–43). In a recent study exploring dance science knowledge, dancer educators identified mental health and psychology as topics requiring more research (44). Furthermore, recent recommendations by Reece et al. (45) state: “Treatment of LEA requires addressing the underlying cause. The gold standard for treatment of disordered eating/eating disorders is a multidisciplinary, collaborative approach. Education for athletes, parents, coaches, and athletic trainers is imperative for the detection and prevention of LEA and disordered eating/eating disorders” (45). Indeed, in the last decade there has been an increase in eating disorder prevention and nutrition education interventions developed for athletes and dancers. Education programmes focused on promoting body acceptance [e.g., the Body Project (46) and the Body Project with dancers (47)] and incorporating behaviour change such as using cognitive-behaviour-dissonance (48) show promise in reducing body dissatisfaction, reducing the risk of eating disorders, and in increasing the number of athlete’s seeking professional advice regarding female triad symptoms (46). Despite progress in health education programmes for dancers [e.g., (47, 49)], addressing education at a broader level including dance teachers and industry, along with a culture shift away from thinness idealisations in dance is required (4, 10). Safeguarding the health and welfare of high-performance athletes has been flagged as a priority in all sporting environments, including among young athletes (21), and the importance of sports medicine within performing arts has been acknowledged (11, 50). Recommendations for principles and functions of health services within Australian high-performance sport have recently been proposed (21) including for disordered eating (51). However, despite the promising progress in elite sport, guidelines for healthcare services within the context of dance are lacking in Australian educational programmes.

### 4.1 Limitations and future research

Several limitations are worth noting. First, the sample only included dancers from one Australian tertiary institution. However, dancers originated from states and territories across Australia with half of all participants in first year of study, and as such, the findings reflect Australian dancers embarking on full-time training. Second, the small number of male participants in this study limited the ability to analyse and compare differences between male and female dancers across all measures. We were, however, able to analyse BMI, LEA, and risk of REDs, with which males were at lower risk than female dancers. Nonetheless, education remains important to promote career longevity among males as it does females (27) and is an important consideration in future research. Third, the DEAQ self-report tool has been validated among dancers previously and in this study it was adapted for the Australian environment. Inclusion of additional health measures such as blood work and bone mineral density such as those outlined in the 2023 IOC REDs Consensus Statement (5) would allow for a greater understanding of LEA and REDs indicators among dancers. Fourth, alcohol intake was not measured in this study. A study of professional dancers has compared weekday energy intake to weekends and dancers do increase their energy intake over weekends from fat and alcohol, reducing carbohydrate and protein when they are not dancing (18). As alcohol influences energy intake, potentially fat mass (52), and macro and micronutrient absorption (53) it is recommended alcohol intake is considered in future studies among dancers. Lastly, the current study was descriptive in nature. To provide greater insight into understanding reasons for certain choices or behaviours (such as supplement use or eliminating specific foods) a range of questions could be used to probe certain issues. Additionally, focus groups or clinical interviews would allow a more comprehensive understanding of the needs of dancers, dance teachers, and those involved in professional companies along with the perceived barriers and facilitators of health and nutrition education in elite dance.

### 4.2 Practical implications

Recognising dancers as athletes may be the first step in helping the dance community move forward on a range of issues related to dancer health and wellbeing. For training institutions, the focus needs to be on ensuring curriculum and education standards reflect current knowledge in dance and sport science as well as facilitating access to a range of health professionals, with health promotion and prevention of injury at the forefront of the dance training philosophy. Evaluation of dancer knowledge of preventative health and correcting misinformation will help break the cycle of misinformation, as today’s pre-professional dancers will likely become the teachers and artistic directors of the future. Screening in a manner that engages the dancer rather than punishing their self-esteem may also be necessary to adequately risk assess and manage the needs of each cohort. Early screening will help facilitate earlier intervention and protect and promote longevity in dance careers. Screening questions could be incorporated into existing physical and health screenings [e.g., (54)] or built into early curriculum to raise dancer self-awareness.

## 5 Conclusion

This research has helped establish an understanding of dance students’ knowledge of current health, nutrition issues, lifestyle, wellbeing, and understanding of longer-term health implications. As a crude indication, one-third of female dancers had a negative REDs risk score. A similar proportion had a BMI < 20, previous fractures, a history of secondary amenorrhea, and had experienced oligomenorrhea. Awareness of REDs and the connection with menstrual issues was relatively low among dancers despite this being a good indicator of overall current physical health. Broad engagement of the dance community is called to action, to acknowledge the need to develop programmes for dancers of all ages and level of training that help protect and empower dancers in relation to health, nutrition, fitness, and wellbeing. This study also highlights the importance of adequate healthcare within dance, including access to relevant healthcare practitioners within elite training environments, along with improved awareness and education of the physical and psychological demands of dancing and LEA and REDs among all practitioners working with dancers.

## Data availability statement

The datasets presented in this article are not readily available because ethical approval and participant consent was not obtained for sharing data. Requests to access the datasets should be directed to JN, j.nicholas@ecu.edu.au.

## Ethics statement

Ethics approval for this study was granted by the Edith Cowan University Human Research Ethics Committee (REMS NO: 2022-03575-NICHOLAS) and University of New South Wales Human Research Ethics Committee. The studies were conducted in accordance with the local legislation and institutional requirements. Where participants were <18 years of age, written informed consent was provided by the participants’ legal guardians/next of kin.

## Author contributions

JN: Conceptualization, Formal analysis, Investigation, Methodology, Writing–original draft, Writing–review and editing. SG: Conceptualization, Formal analysis, Investigation, Methodology, Writing–original draft, Writing–review and editing.
